# Clinical Management of Root Resorption: A Report of Three Cases

**DOI:** 10.7759/cureus.3215

**Published:** 2018-08-27

**Authors:** Neha Mehra, Mona Yadav, Mamta Kaushik, Roshni Roshni

**Affiliations:** 1 Conservative Dentistry and Endodontics, Army College of Dental Sciences, Secunderabad, IND; 2 Private Practice, Clove Dental Clinic, Gurgaon, IND; 3 Conservative Dentistry and Endodontics, Army College of Dental Sciences, Hyderabad , IND

**Keywords:** internal root resorption, perforating resorption, invasive cervical resorption, cone beam computed tomography (cbct), mineraltrioxide aggregate (mta), biodentine

## Abstract

Root resorption is a pathological condition that may be an endodontic challenge if not diagnosed and treated correctly. The recent advances in the imaging technologies and material science have enabled the clinician to visualize the structural changes accurately and repair them with materials providing favourable seal ability.

In this article, we report three cases of root resorption with different presentations which were diagnosed with the help of cone beam computed tomography (CBCT) and successfully managed. The series highlights the importance of diagnosis in unusual clinical situations and recommends early commencement of optimal management for longevity of tooth for health and function.

## Introduction

Root resorption, a physiologic process in deciduous teeth, is a pathological condition if encountered in permanent teeth. This may result in loss of tooth, if not diagnosed and treated correctly [1].

The process of tooth resorption is a highly structured interaction amongst inflammatory cells, resorbing cells (osteoclasts, odontoclasts or dentinoclasts) and hard tissues, initiated by injury to the non-mineralized tissues covering the external surface of root (precementum) or the internal surface of the root canal (predentin) [2,3]. The transformation of precursor cells into clastic cells is induced by cytokines, of which interleukin-1𝛽 plays a pivotal role [4].

Resorption can broadly be classified as External resorption, Internal resorption or both depending upon the location of the process.

Internal root resorption (IRR), commonly referred to as intracanal resorption, is a rare occurrence resulting in dystrophy of the pulp that leads to destruction of the hard tissues, leading to morphological changes. Its prevalence ranges between 0.1 and 1.6% [1]. Majority of the cases remain asymptomatic and are often incidentally detected in radiographs. Once detected, it should be treated as soon as possible to limit its progression.

External root resorption could be surface, inflammatory, replacement, and cervical resorption. External root resorption is more common than internal resorption and is frequently confused radiographically as internal resorption.

Invasive cervical resorption (ICR) is a clinical condition which exhibits a relatively uncommon and often aggressive form of external tooth resorption. Its cervical location and invasive nature, lead to progressive and extensive loss of tooth structure [5]. The diagnosis and treatment of ICR depends on the extent of resorption into the dentin. Treatment options include accessing the defect, cleaning and restoring it with either the placement of a suitable filling material or by the use of biological systems [6]. The treatment of advanced ICR is challenging, and has poor prognosis.

This paper insights a varied series of management and follow-up of three cases of resorption with satisfactory healing.

## Case presentation

Case 1: Inflammatory non-perforating internal root resorption

A 35-year-old healthy female patient presented to the Department of Conservative Dentistry and Endodontics, Army College of Dental Sciences, Secunderabad, India, with dull pain in right upper front tooth region for one week. The patient gave a history of trauma 10 years ago, and previous dental treatment with respect to the same tooth.

**Figure 1:**
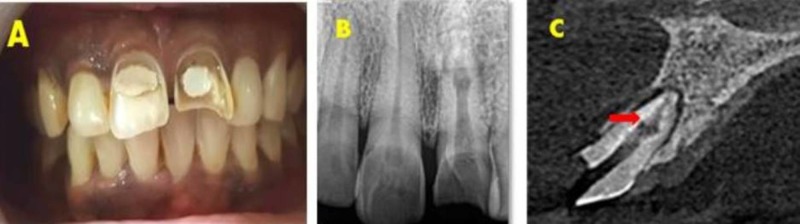
Preoperative assessment for patient with inflammatory non-perforating internal root resorption. (A) Pre-operative photograph. (B) Pre-operative radiograph. (C) CBCT image showing sagittal view of tooth 21 with an internal resorptive defect. CBCT: Cone beam computed tomography

Clinical examination revealed previously initiated endodontic therapy in tooth 11 and 21 (Figure 1A). Tooth 21 showed mild sensitivity to percussion with no associated sinus formation or swelling. Radiographic examination revealed a well-defined radiolucency in the middle and apical third of tooth 21 (Figure 1B).

Cone beam computed tomography (CBCT) (Kodak 9500 Cone Beam 3D system, USA) was done using field of view 5 × 5 and axial, sagittal, and horizontal sections were obtained that aided in the diagnosis of inflammatory non-perforating internal resorption with symptomatic apical periodontitis for tooth 21 (Figure 1C) and chronic irreversible pulpitis with normal periapical tissues for tooth 11. Nonsurgical endodontic treatment was planned in relation to tooth 21 and 11.

First Appointment

**Figure 2:**
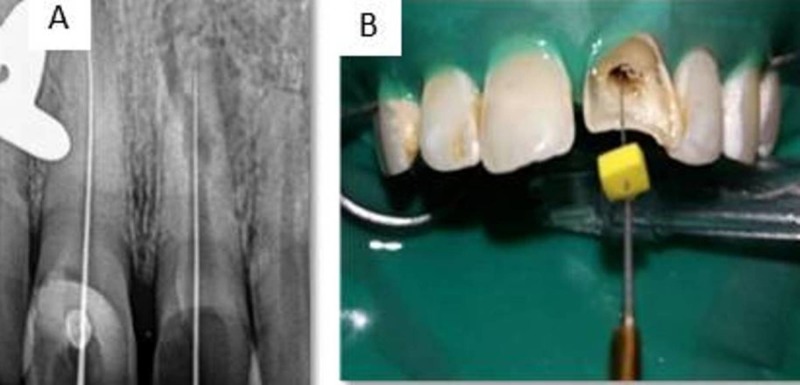
Treatment procedure on first appointment. (A) Working length radiograph for tooth 11 and 21. (B) Ultrasonic irrigation performed on tooth 21.

Informed consent was obtained from the patient and treatment initiated by administering an infiltration of 2% Lignocaine with 1:80,000 adrenaline (Lignox, Indoco Remedies Ltd, India). The tooth was isolated using rubber dam (Hygenic Dental Dam, Coltene Whaledent, Germany) and coronal access was prepared using an Endo‑Access bur (Dentsply Maillefer, USA). The working length was determined using apex locator (Root ZX II; Morita, Tokyo, Japan) (Figure 2A) which was found to be 23 mm in tooth 11 and 20 mm in tooth 21.

Cleaning and shaping was performed by crown-down technique using Protaper Universal (Dentsply Maillefer, USA) up to finishing file number 3 (F3), under copious irrigation using 2.5% sodium hypochlorite (Septodont, India) and normal saline. Ultrasonic irrigation using Irrisafe ultrasonic tips (Satelec, France) (Figure 2B) was performed with 2.5% sodium hypochlorite and an intracanal dressing of calcium hydroxide was given and temporarily sealed using Cavit (3M ESPE, USA).

Second Appointment

On two-week recall visit, the intracanal medicament was removed using 10% citric acid (Prime Dental Products Pvt Ltd, Maharashtra, India) using ultrasonic irrigation. The tooth was asymptomatic, and prepared for obturation.

**Figure 3:**
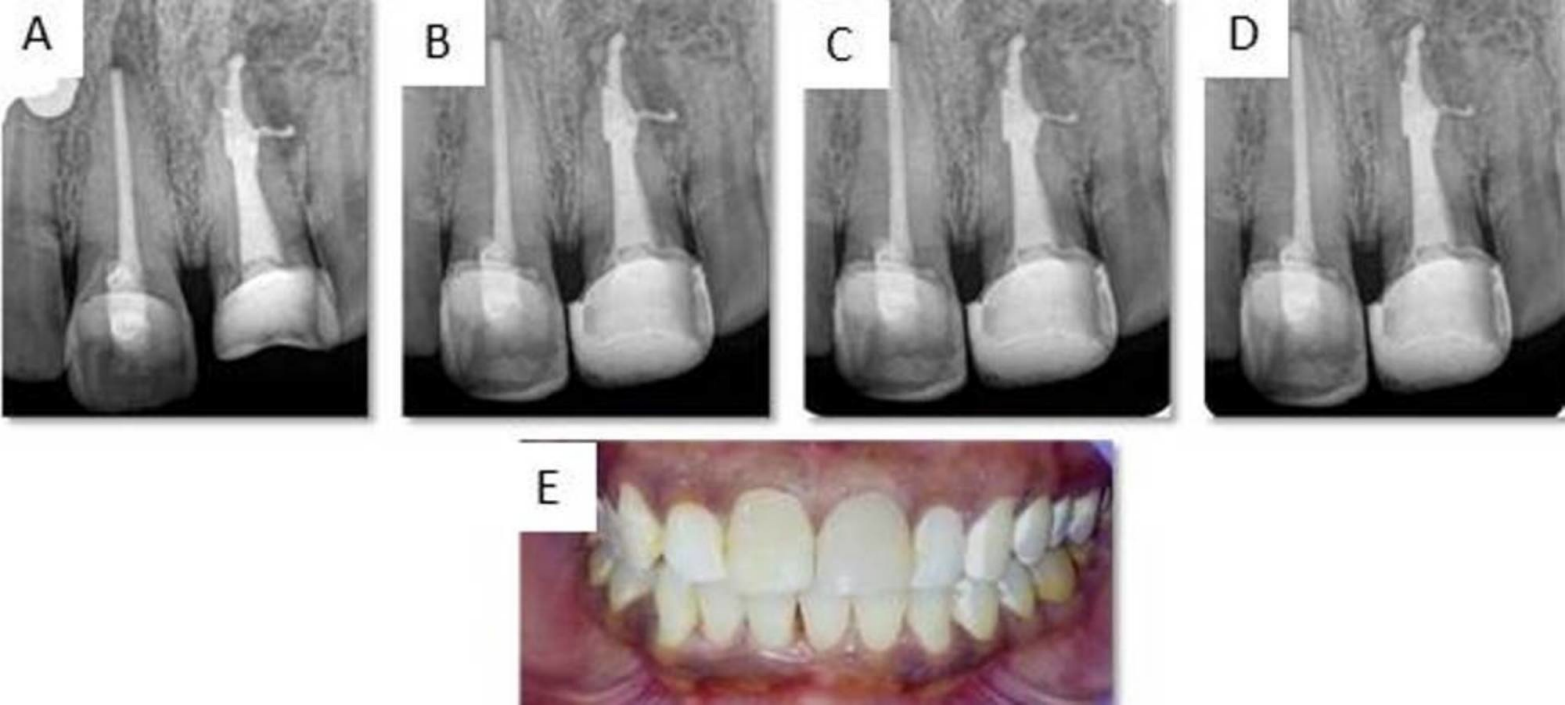
Postoperative radiograph and photograph of tooth 11 and 21. (A) Obturation. (B) Six-month follow-up. (C) 12-month follow-up. (D) 18-month follow-up. (E) Post operative photograph.

Since the resorptive defect involved the middle and apical region of tooth 21, the entire canal was obturated with thermoplasticized gutta-percha, using Obtura II (Obtura, USA) while tooth 11 was with lateral compaction technique using gutta-percha (Dentsply Maillefer, USA) and AH Plus (Dentsply Maillefer, USA) (Figure 3A). The tooth was clinically asymptomatic and showed successful healing radiographically at six, 12 and 18 months (Figure 3B, 3C, 3D). Figure 3E shows the postoperative photograph of patient with satisfactory healing.

Case 2: Inflammatory perforating internal root resorption

**Figure 4:**
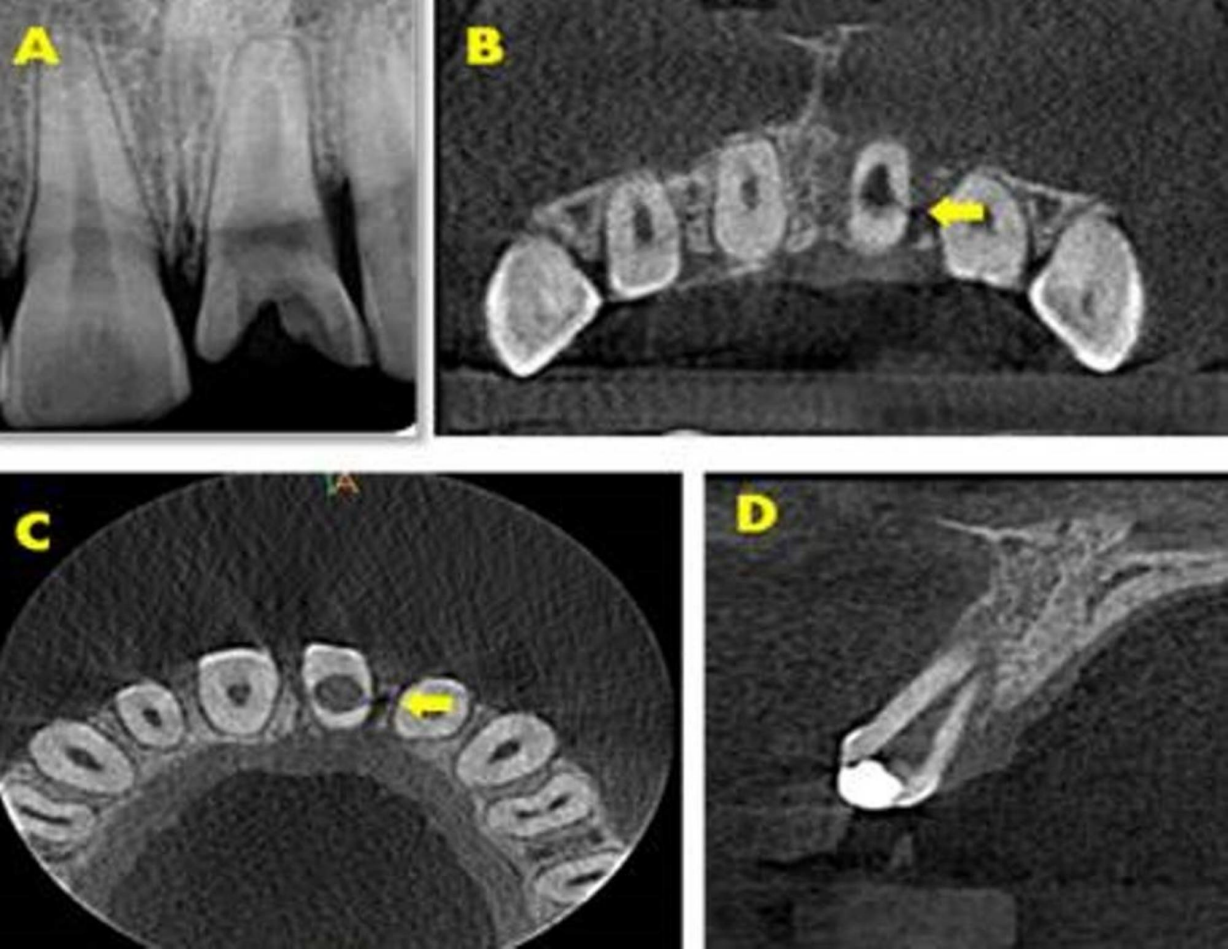
Preoperative assessment for inflammatory perforating internal root resorption. (A) Preoperative radiograph with enlargement of root canal space suggesting internal root resorption. (B) Axial section of CBCT showing communication with external root surface. (C) Axial section of CBCT with internal root resorption and perforating with external root in the coronal third. (D) Sagittal section showing enlargement of root canal suggesting internal root resorption. CBCT: Cone beam computed tomography

A 20-year-old male patient presented with pain for one week and gave a history of trauma seven years back leading to fracture of tooth 21. Clinically, the tooth 21 showed grossly destructed crown, a 6 mm deep periodontal pocket distally and grade one mobility. Intra oral periapical radiograph revealed a well-defined radiolucency in the coronal third of the radicular surface (Figure 4A). Images of cone beam computed tomography revealed a radiolucency communicating with the external root surface, suggestive of inflammatory perforating internal root resorption (Figure 4B, 4C, 4D). Since the prognosis of the tooth was questionable, extraction was the treatment of choice, but the patient desired to save the tooth.

First Appointment

**Figure 5:**
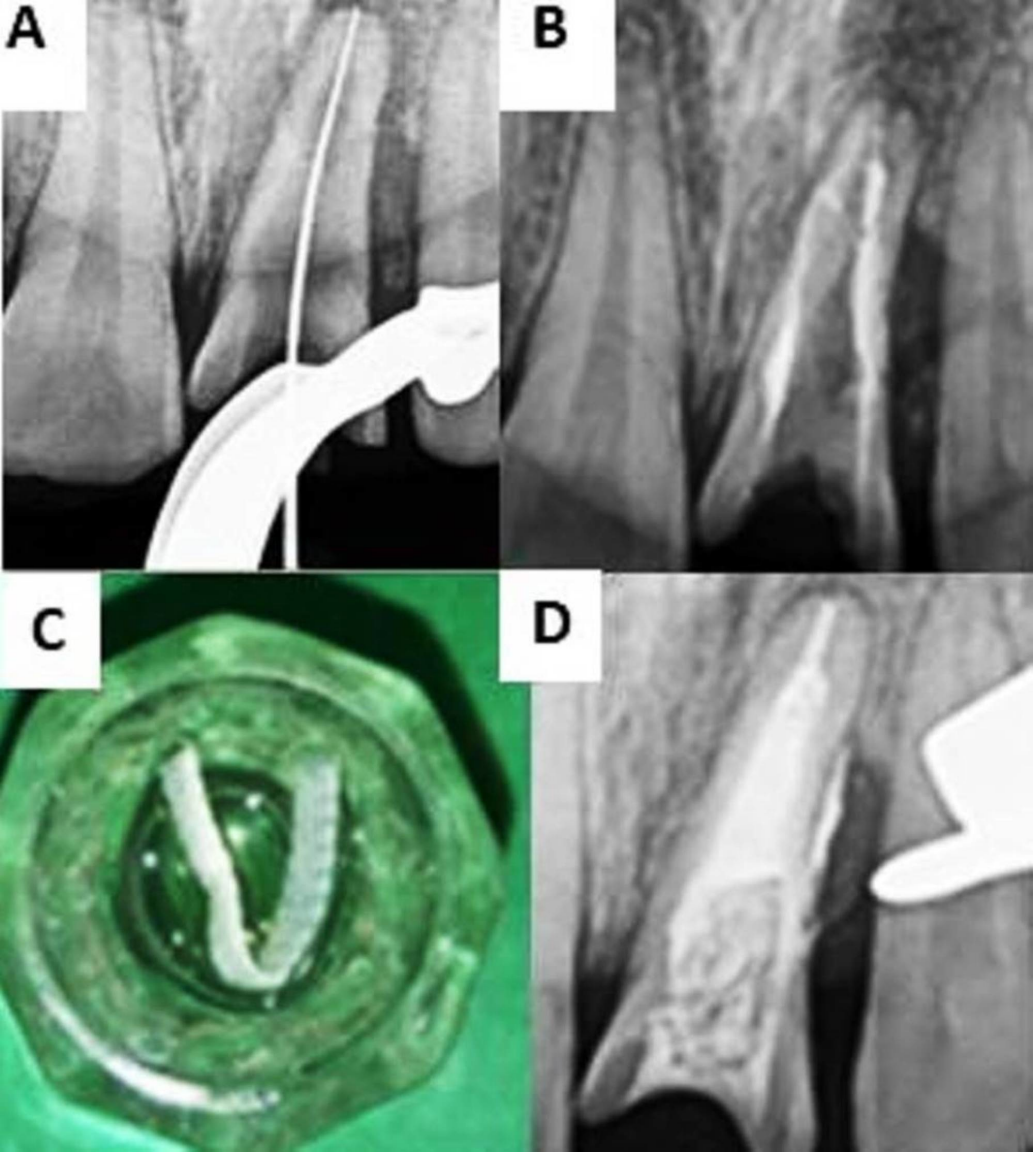
Treatment procedure for tooth 21. (A) Working length determination. (B) Repair of perforation internally using MTA. (C) Ribbond fibres. (D) Post-obturation radiograph. MTA: Mineral trioxide aggregate

An informed consent was obtained and root canal was accessed after administration of 2% local anaesthesia with 1:80,000 adrenaline under rubber dam isolation, using an Endo‑Access bur. The intracanal bleeding was controlled by gently irrigating with 2.5% sodium hypochlorite. Working length of 23 mm was established (Figure 5A) and cleaning and shaping was performed with Protaper Universal up to finishing file F3. Calcium hydroxide (Prime Dental Pvt Ltd, India) was placed for a period of four weeks as an intracanal medicament and temporized with Cavit.

Second Appointment

Calcium hydroxide dressing was removed with 10% citric acid, using ultrasonic irrigation and internal walls of the canal were repaired with mineral trioxide aggregate (MTA) (Angelus, Brazil), sealed with a wet cotton pellet and temporized with Cavit, and allowed to set for 24 hours (Figure 5B). Sectional obturation was done followed by backfill with thermoplasticized gutta-percha, using Obtura II, till the middle third of the canal. The remaining canal was sealed with Ribbond fibres (Ribbond, USA) (Figure 5C) and composite (Ivoclar Vivadent, NY, USA). Figure 5D shows the postoperative radiograph of tooth 21 after obturation.

**Figure 6:**
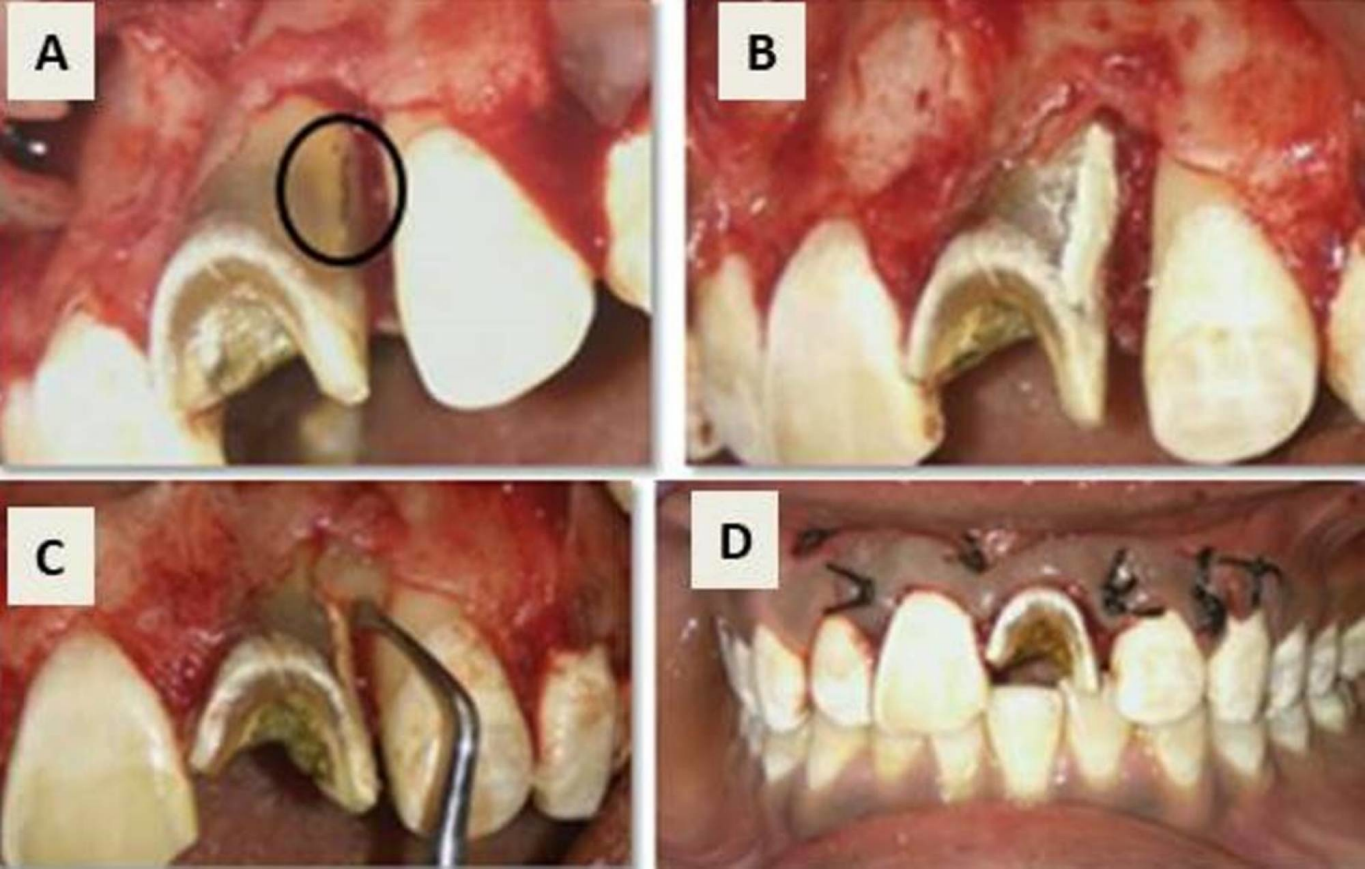
Surgical repair of the resorptive defect. (A) Perforation accessed surgically. (B) Perforation repair done using mineral trioxide aggregate (MTA). (C) Placement of bone graft and platelet rich fibrin (PRF) membrane. (D) Sutures placed.

A full thickness mucoperiosteal flap was reflected (Figure 6A) and the perforation defect was repaired externally using MTA (Figure 6B). The associated periradicular bone defect was curetted and biogen granules (Biotek, Italy) were packed into the defect with platelet rich fibrin (PRF) membrane (Figure 6C) and sutured (Figure 6D). The patient was recalled at one week, four weeks, six and 18 months for follow-up.

**Figure 7:**
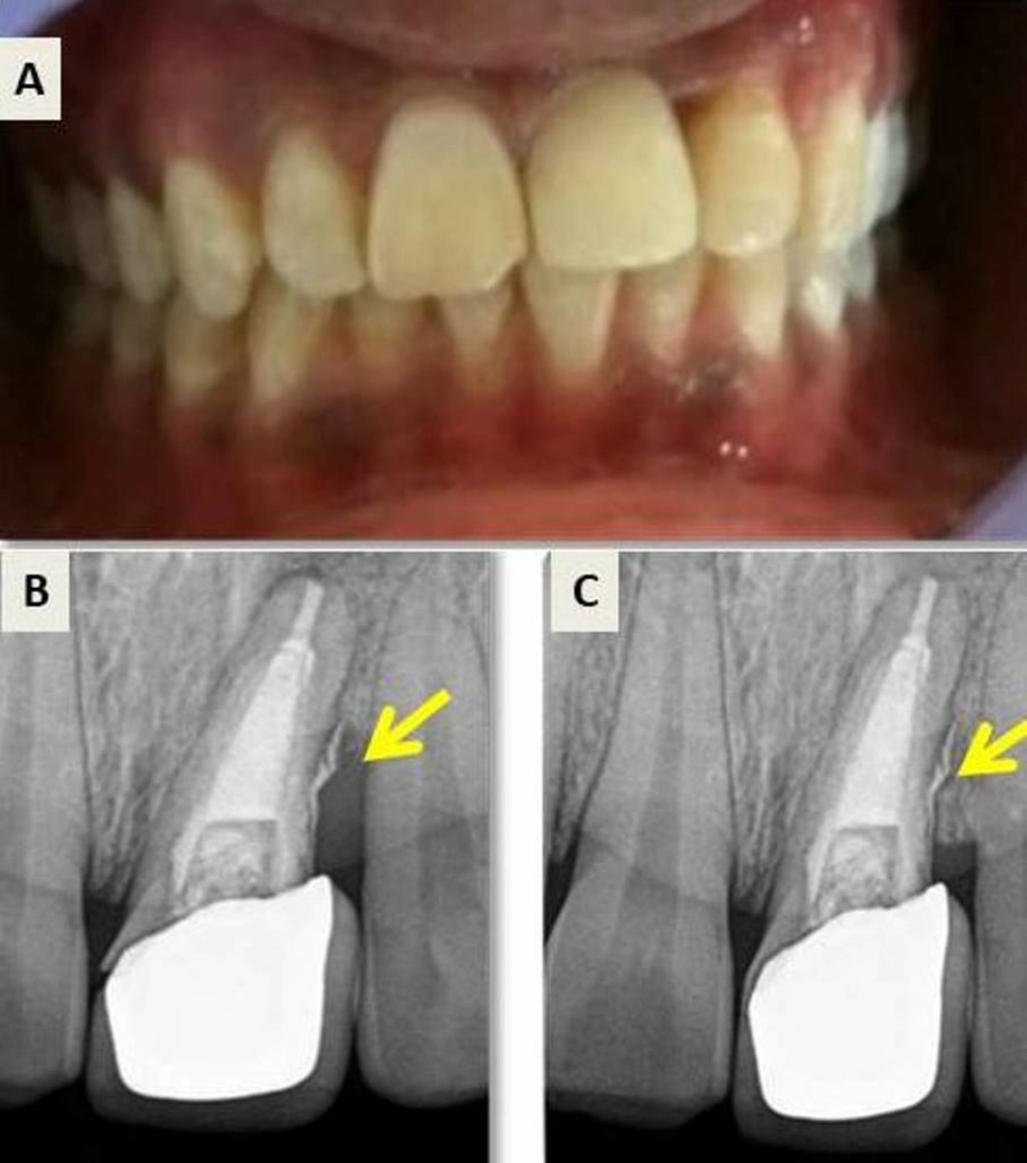
Postoperative photograph and follow-up radiograph. (A) Post-operative photograph of the patient. (B) Six-month follow-up. (C) 18 months follow-up showing healing with bone formation.

Figure 7 depicts the post-operative clinical photograph and six and 18 months recall visit radiographs, showing healing of the lesion with bone formation.

Case 3: Invasive cervical external root resorption

**Figure 8:**
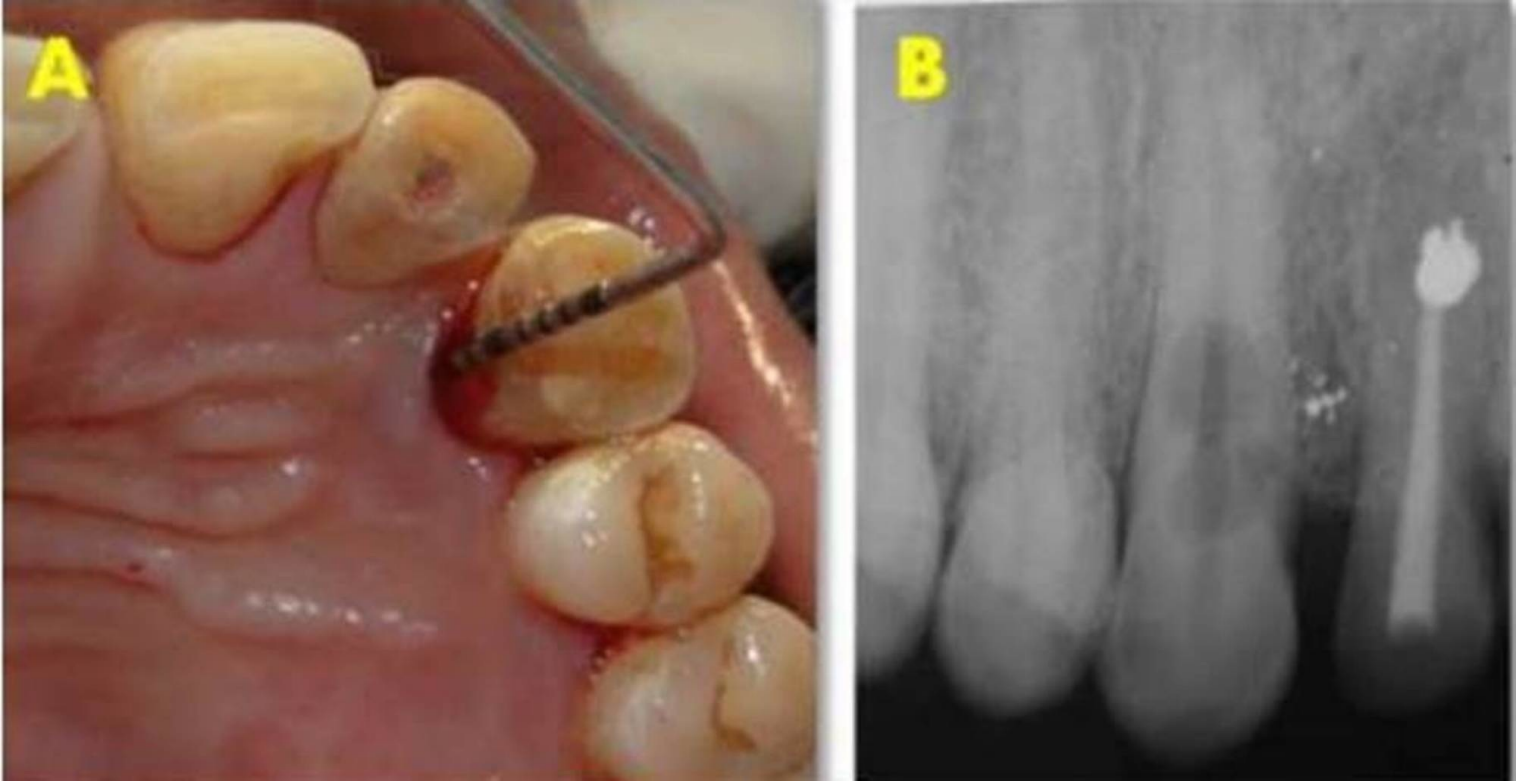
Preoperative clinical and radiographic assessment. (A) Deep periodontal pocket in tooth 23 present lingually. (B) Preoperative radiograph.

A 43-year-old male patient presented with pain in left upper quadrant from the past one month. The patient gave a history of trauma 12 years back for which surgery was performed on the left upper lateral incisor 10 years ago. Clinically, tooth 22 was asymptomatic; however, the tooth 23 presented with an intraoral sinus and showed moderate sensitivity to percussion. Cold test and electric pulp test revealed a delayed response for tooth 23. An 8 mm deep periodontal pocket was present on the palatal surface of tooth 23 (Figure 8A). Radiographic analysis revealed diffuse radiolucencies in cervical and mid root region of tooth 23 and root canal treated 22 (Figure 8B).

**Figure 9:**
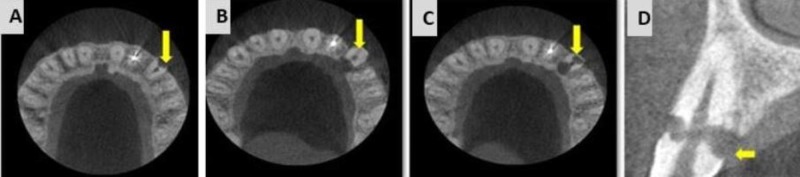
Cone beam computed tomography (CBCT) analysis for tooth 23. (A) CBCT scan: Axial section of tooth 23, showing cervical root resorption, labially. (B) Axial section showing invasive cervical root resorption at cervical level, lingually. (C) Axial section showing invasive cervical root resorption at cervical level, labially and lingually. (D) Sagittal section showing, class III invasive cervical resorption.

To obtain specific knowledge about the three-dimensional (3D) anatomy, the patient was referred for a CBCT analysis. CBCT revealed a class 3 cervical root resorption on buccal and palatal aspects, which perforated and involved the main canal (Figure 9A, 9B, 9C, 9D).

First Appointment

After obtaining an informed consent the tooth was isolated using rubber dam, access cavity was prepared in tooth 23 using an Endo‑Access bur. Working length of 25 mm was established using apex locator. Cleaning and shaping of canals was performed followed by placement of calcium hydroxide for a period of four weeks, and temporized with Cavit.

Second Appointment

**Figure 10:**
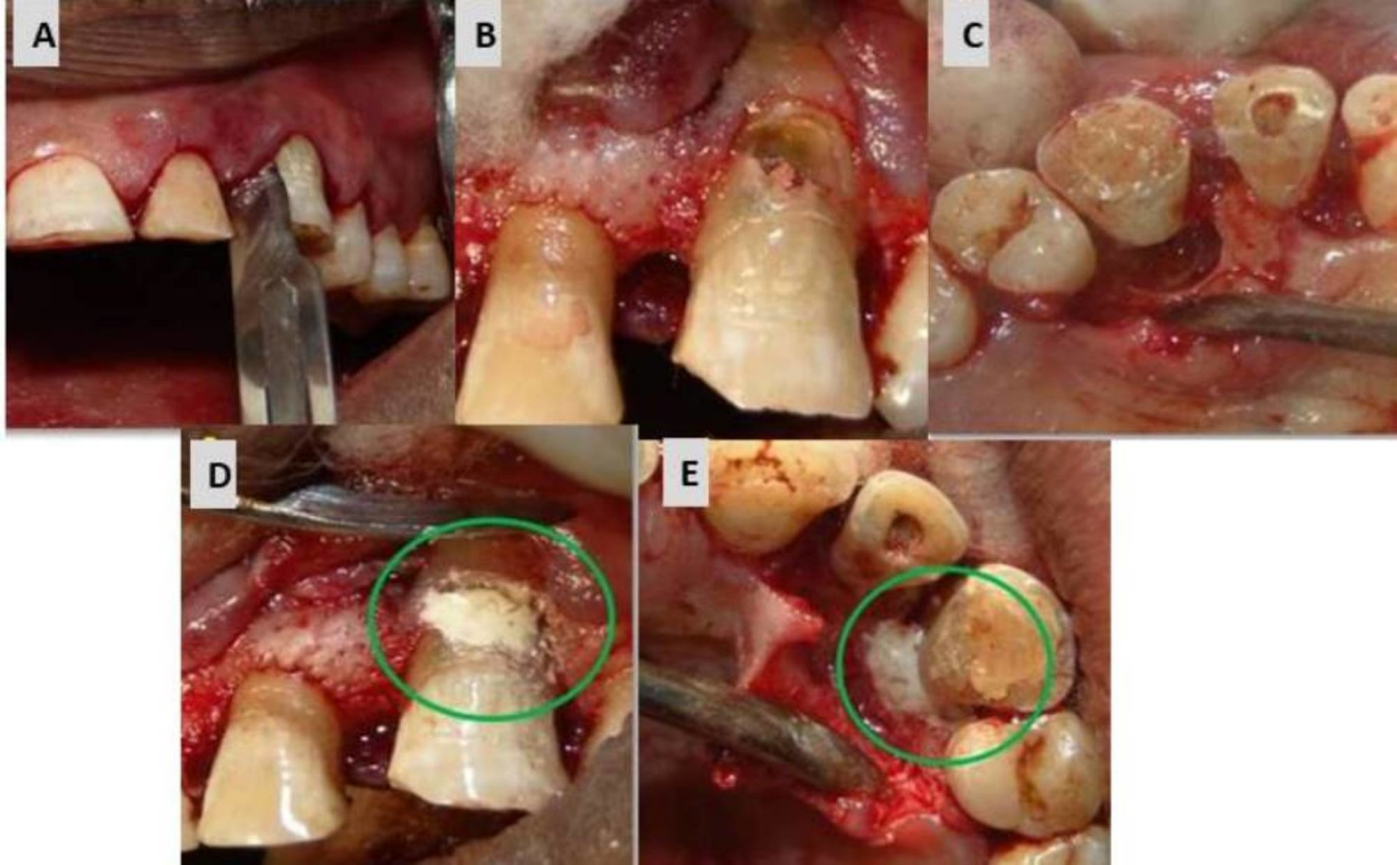
Surgical repair of the resorptive defect with biodentine. (A) Sulcular incision. (B) Resorption defect exposed labially. (C) Resorption defect exposed lingually. (D) Resorption defect restored with biodentine labially. (E) Resorption defect restored with biodentine lingually.

At the recall visit, medicament was removed using 10% citric acid. A surgical intervention was planned to make the cervical defect accessible. A triangular full thickness flap was reflected buccally and palatally to expose the large defect (Figure 10A). The necrotic tissue was curetted, and resorptive defect was cleaned with normal saline and 17% ethylenediaminetetraacetic acid (EDTA) (Prime Dental product Limited, India) (Figure 10B, 10C). The root canal was dried using paper points (Diadent, USA) and secured by inserting a 2% gutta-percha point (Diadent, USA). The defect was repaired using Biodentine (Septodont, USA) on both buccal and palatal surfaces and contoured according to the external anatomy of the root (Figure 10D, 10E).

**Figure 11:**
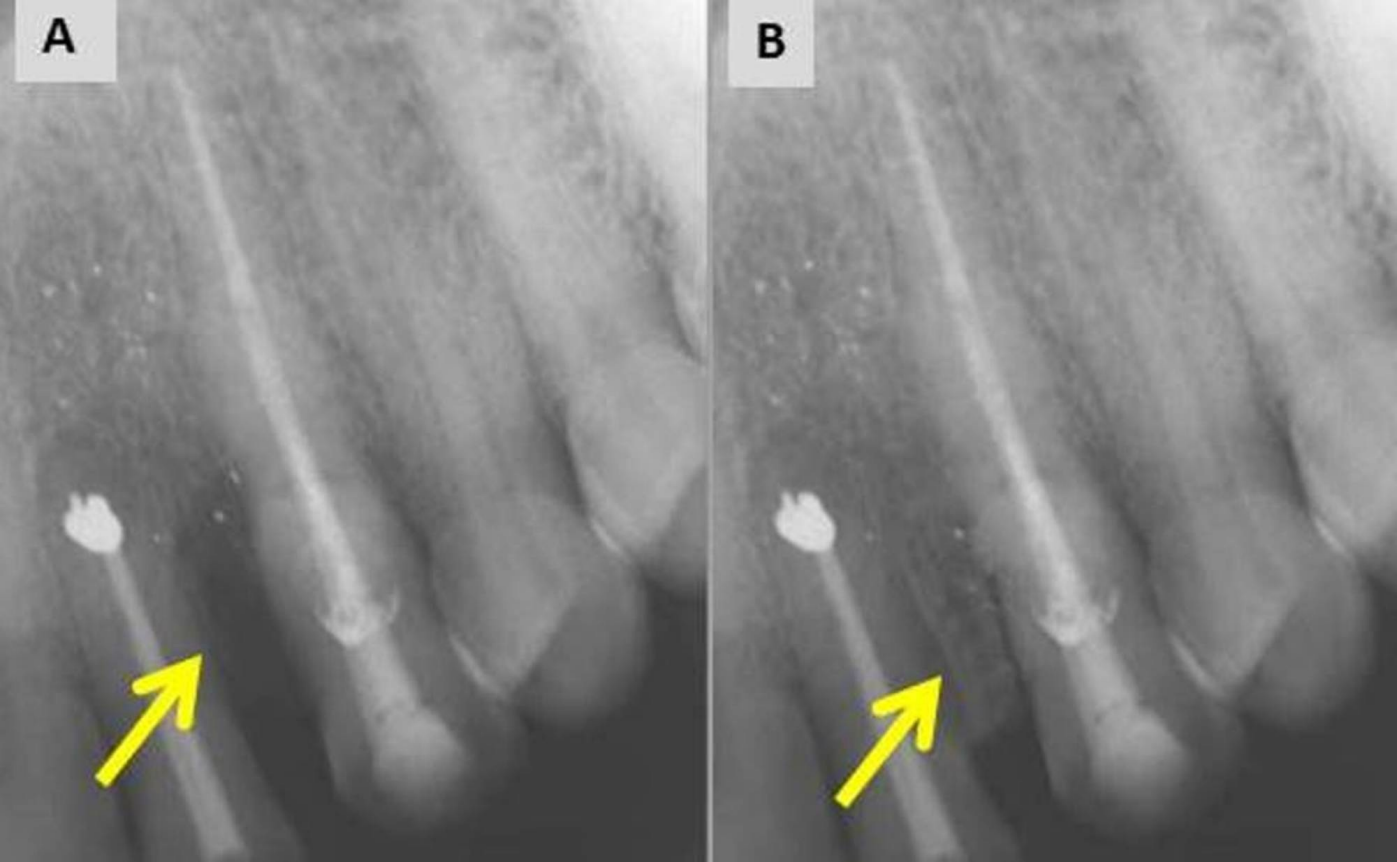
Postoperative and follow-up radiograph. (A) Post-operative radiograph. (B) Four-year follow-up radiograph showing satisfactory healing with bone formation.

Sectional obturation was done followed by backfill using thermoplasticized gutta-percha and MTA Fillapex (Angelus, Brazil). The flap was repositioned and sutured. The patient was instructed to report after a week for suture removal. A four-year follow-up radiograph shows satisfactory periradicular healing with bone formation (Figure 11A, 11B).

## Discussion

In endodontic practice, root resorption is one of the commonly encountered challenges. Early detection and accurate differential diagnosis are important factors that determine the successful clinical outcome of cases with resorption.

It is important for the diagnostician to differentiate internal resorption from external tooth resorption. Radiographs and knowledge of internal root anatomy help a lot in these matters. With the development of technology and CBCT as diagnostic aids the accurate diagnosis and predictable treatment and prognosis.

If the tooth is restorable and has a reasonable prognosis, root canal is the treatment of choice. The aim of root canal treatment is to remove all vital and necrotic tissue, to ensure that the resorbing cells are lysed and to disinfect and obturate the root canal system [7].

The complexities of the root canal system and inaccessibility of the defect offer technical difficulties for thorough disinfection of the root canal, thus a chemo-mechanical approach along with an intracanal antibacterial medicament is advocated in such cases to render the canal bacteria free [7].

In cases with perforating defects, the resorptive cavity is sealed with calcium silicate-based materials that form a hard tissue barrier against which the obturating material is condensed [7]. In some cases where the defect is inaccessible a surgical approach is recommended and where there is extensive defect, extraction may be indicated [8]. The present case series discusses the alternate treatment modalities for different presentations of root resorption.

Case report 1 presents a non-surgical approach for an inflammatory non-perforating internal root resorption obturated using thermoplasticized gutta-percha after thorough debridement and disinfection of root canal.

Cases 2 and 3 that presented with extensive resorptions were treated by a hybrid technique, where after chemomechanical debridement, the defects were sealed surgically with MTA and Biodentine, respectively. These materials are biocompatible [9], have good sealing property [10], well tolerated by the periodontal tissues and allow a complete regeneration of the periodontium [11]. Following the repair, the root canals were obturated with thermoplasticized gutta-percha to form a 3D seal.

Studies have reported that traditional radiography does not always reliably reveal the presence of a lesion and does not show the real size, extent of the lesion and its spatial relationship with anatomic structures. On the contrary, 3D imaging allows us to visualize the third dimension while eliminating superimpositions of surrounding anatomic structures and provides geometrical accuracy of linear measurements [12].

In the present case series, 3D imaging by CBCT has helped in assessment of the resorptive defects by predicting the treatment complexity and expected outcome based on the location and extension of the defect.

## Conclusions

Timely diagnosis, removal of the cause and proper treatment are obligatory for successful treatment outcome. With the advent of superior diagnostic technologies like, CBCT, a more conservative management can be expected. Modern endodontic techniques including optical aids, ultrasonics, and thermoplastic filling techniques and use of materials such as MTA and Biodentine offer opportunities for rehabilitation of the resorbed teeth.
